# A giant biliary cyst of Todani IA in a young woman: A case report

**DOI:** 10.1097/MD.0000000000024022

**Published:** 2021-01-15

**Authors:** Sławomir Mrowiec, Beata Jabłońska, Jan Baron, Magdalena Gajda, Anna Stelmach, Patryk Zemła, Łukasz Liszka

**Affiliations:** a Department of Gastrointestinal Surgery; b Department of Radiology; c Student Scientific Society, Department of Gastrointestinal Surgery; d Department of Pathomorphology and Molecular Diagnostics, Medical University of Silesia, Katowice, Poland.

**Keywords:** bile duct, biliary cyst, hepaticojejunostomy

## Abstract

**Rationale:**

Biliary cysts (BC) are rare dilatations of various parts of a biliary tract. They account for approximately 1% of all benign biliary diseases. Total cyst excision and Roux-Y hepaticojejunostomy is the treatment method of choice in most patients. In this paper, a novel surgical treatment with the use of internal biliary and pancreatic catheters was presented.

**Patient concerns:**

A 21-years-old woman with a giant choledochal cyst of Todani IA type presenting with abdominal pain, nausea, fever and palpable abdominal mass. It had been previously drained as a misdiagnosed pancreatic cyst in another hospital.

**Diagnosis:**

A very high amylase level (107140,0 U/l) in drain fluid was noted in laboratory tests. Endoscopic retrograde cholangiopancreatography revealed a biliary cyst located in the distal common bile duct and a pancreaticobiliary anomaly was suggested. A cholangiography per catheter inserted to the biliary cyst showed a large round contrast-filled cyst. A cholangiography following cyst decompression revealed a very long, tortuous bile duct entering the duodenum.

**Interventions:**

Cholecystectomy, cyst resection, Roux-Y hepaticojejunostomy, and implantation of catheters into pancreatic and bile duct were performed. The postoperative course was uneventful and she was discharged on 12th day without any complications. Histopathology revealed a cyst wall partially lined with biliary-type and mucinous epithelium, with dysplasia ranging from low to high grade (biliary intraepithelial neoplasia, high grade), without invasion.

**Outcomes:**

The biliary and pancreatic catheters were removed during endoscopic retrograde cholangiopancreatography 8 weeks following surgery without any complications. Fourteen months later, the patient reported good health.

**Lessons:**

Diagnosis of the abdominal cyst should be very precise in order to avoid misdiagnosis and inadequate management. The early diagnosis and proper treatment of BC are needed in order to avoid serious complications. The cholangiocarcinoma is the most dangerous potential complication of BC due to dysplasia within the cyst wall as in our young female patient.

## 1 Introduction

Biliary cysts (BC) (1% of all benign biliary diseases) are rare dilatations of different parts of a biliary tract (BT).^[1]^ The incidence of these congenital disorders is 1/2000000 live births.^[2]^ They have higher prevalence in the Asian population compared to Western population.^[3]^ Commonly, BC are manifested with a right subcostal pain, abdominal palpable mass and jaundice in infancy or childhood. In 20% of patients they are diagnosed in adults.^[1]^ The early diagnosis and treatment are important, due to a risk (2.5%–28%) of carcinogenesis in BC.^[1,4]^

According to Babbitts's theory of the “common channel”, the common cannel is formed by abnormal pancreaticobiliary junction of the pancreatic and bile ducts outside the ampulla of Vater.^[5,6]^ It leads to pancreaticobiliary reflux and mixing of pancreatic and biliary juices and activation of pancreatic enzymes, because the pressure within the pancreatic duct (PD) is 2 to 3 timed higher than within BT. Activated pancreatic enzymes cause inflammation and deterioration of the bile duct wall that leads to biliary dilatation.^[1,5]^ Association between the high amylase level, earlier presentation and dysplasia grade has been reported. The pancreaticobiliary reflux leads to inflammation and dysplasia.^[1]^

BC are divided into 5 types according to Todani classification.^[7]^ Additionally, type VI (including BC of the isolated cystic duct) has been described.^[8]^ BC need surgery to avoid complications associated with pancreaticobiliary reflux. It depends on the cyst type. Total BC excision (with cholecystectomy) and Roux-Y hepaticojejunostomy is the treatment method of choice in most patients.^[1,9]^

## 2 Case presentation

A 21-years old woman was admitted with a history of subcostal pain, fever, nausea, and palpable mass. She had been already after external Petzer drainage (as a misdiagnosed pancreatic cyst) of volume 1000 ml per day in the other hospital. A very high drain fluid amylase level (107140,0 U/l) was noted in laboratory tests.

**Figure 1:**
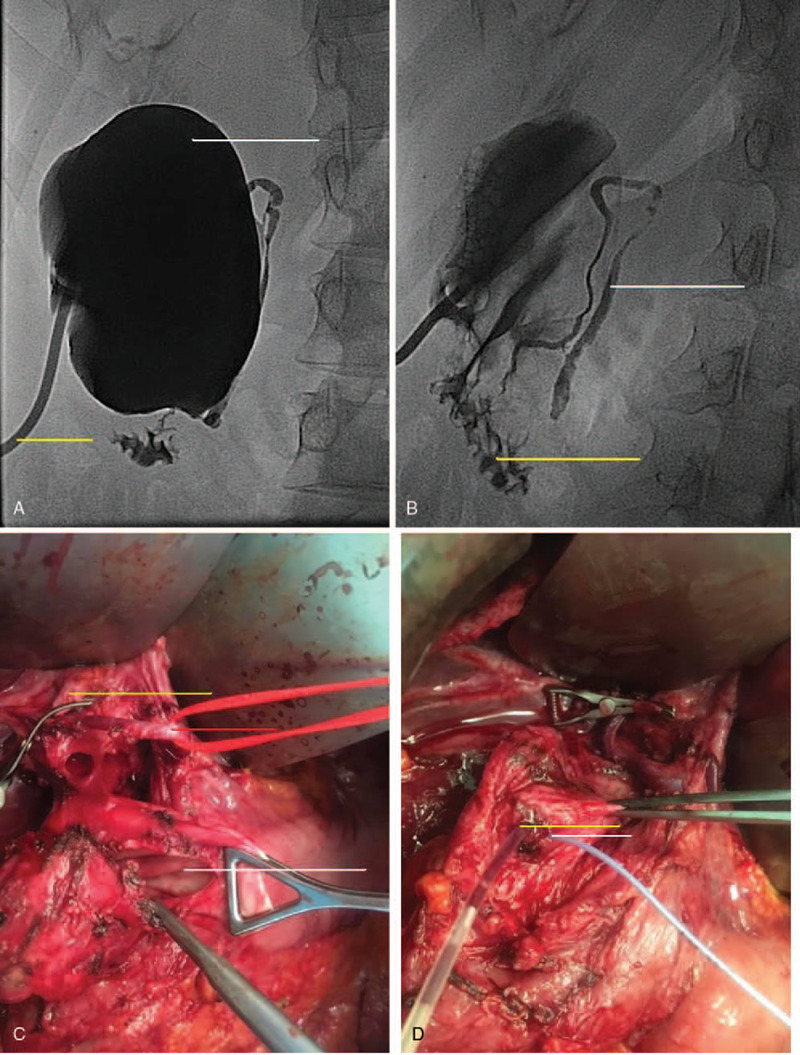
(A) Cholangiography per catheter inserted to the biliary cyst showing a large round contrast-filled cyst. (B) Cholangiography following cyst decompression demonstrating a very long, tortuous bile duct entering the duodenum. (C) Intraoperative picture – a biliary cyst originating from the common bile duct and decompressed gallbladder. (D) Intraoperative picture – after opening of the cyst: 2 channels in the common canal, which were revised using catheters: 1 – leading to the long tortuous distal common bile duct, 2 – leading to the pancreatic duct. An abnormal pancreaticobiliary junction (APBJ) was recognized – junction of the common bile duct and pancreatic duct outside the duodenum.

Endoscopic retrograde cholangiopancreatography (ERCP) revealed a biliary cyst located in the distal common bile duct (CBD) and a pancreaticobiliary anomaly was suggested. Percutaneous transhepatic cholangiography revealed a short (1.5 cm) common hepatic duct and a biliary cyst. A cholangiography per catheter inserted to the biliary cyst showed a large round contrast-filled cyst (Fig. 1A). Cholangiography after cyst decompression revealed a very long, tortuous bile duct entering the duodenum (Fig. 1B).

Laparotomy showed a biliary cyst originating from CBD (Todani IA type) and decompressed gallbladder (Fig. 1C). The cyst was dissected and opened. The Petzer catheter was removed. Cholecystectomy and total cyst resection were performed. After opening of the cyst, 2 channels were revealed in the common canal, which were revised using catheters: 1 – leading to the long tortuous distal CBD, 2 – leading to PD (Fig. 1D). Abnormal pancreaticobiliary junction was recognized. Due to long tortuous CBD and possible communication with the accessory bile duct before opening to the duodenum, implantation of CBD and PD to the duodenum using internal catheters was performed. Roux-Y hepaticojejunostomy was performed.

Histopathology revealed a cyst wall partially lined with biliary-type and mucinous epithelium, with dysplasia ranging from low to high grade (biliary intraepithelial neoplasia, high grade), without invasion. The patient was discharged on 12th postoperative day and the internal catheters were removed during ERCP 8 weeks following surgery. Fourteen months later, the patient reported good health. The patient is still under careful follow-up.

## 3 Discussion

Although BC are congenital bile duct malformations, in 20% patients they are diagnosed in adults, as in our case. According to literature, BC occur 3 times as frequently in women than men.^[10]^ Clinical presentation subcostal pain, fever, nausea, and palpable mass) in our patient was comparable with the literature reports. The classic triad of clinical symptoms was described by Alonso-LEJ et al. It involves right subcostal pain, palpable abdominal mass and jaundice.^[1,11]^ All clinical signs are observed only in 20% to 30% patients.^[12]^ According to Lopez et al, pain is the commonest clinical symptom in adults.^[1,13]^ Recurrent cholangitis and pancreatitis are complications of BC due to pancreaticobiliary reflux.^[1,12,14]^ The other complications observed commonly in adults are following: cholangiocarcinoma, cholecystolithiasis and cholecystitis, liver cirrhosis, cyst perforation.^[1,2,6,14,15]^ Cholecystolithiasis and cholecystitis are caused by the bile stasis in patients with BC. The secondary liver cirrhosis leads to various complications associated with portal hypertension such as upper gastrointestinal bleeding, splenomegaly and pancytopenia.^[1]^ Portal hypertension can also occur without liver cirrhosis due to mechanical obstruction of the portal vein by the cyst.^[1]^ A spontaneous BC rupture occurs in about 1%–12% of patients. It presents with abdominal pain, peritonitis signs and sepsis.^[1]^

Diagnosis of BC involves laboratory and radiological examinations. Laboratory investigations include liver function and cholestasis tests (serum bilirubin, alkaline phosphatase, γ-glutamyltranspeptidase, alanine and aspartate aminotransferases), and amylase levels. The increased amylase concentration in patients with BC has been described in some reports similarly to our case. Also, association between the amylase level, earlier presentation and dysplasia grade in patients with BC has been reported in the literature.^[1,16,17]^ There is a theory, that the pancreaticobiliary reflux leads to inflammation and dysplasia in patients with BC.^[1,18–20]^ Our case confirms this theory, because dysplasia was recognized in the young woman. Radiological imaging is the most useful in BC diagnostics such as ultrasonography of the abdominal cavity, computed tomography of the abdominal cavity, and cholangiography. ultrasonography and computed tomography had been already performed in the other surgical center and the pancreatic cyst was diagnosed based on imaging. In our patient, ERCP and PCT were performed in order to show BT. Cholangiography following cyst decompression was the most useful investigation in our patient, because it revealed a very long, tortuous bile duct entering the duodenum which was not shown in the other investigations.

Intraoperatively, the type IA cyst was recognized. According to literature, the type I biliary cyst is the most frequent (more than 90%). According to Todani classifications, the type IA cyst involves dilatation of the common bile duct, with marked dilatation of part or all of the extra-hepatic bile ducts.^[1,7]^ Early surgical treatment in order to prevent further complications is recommended. Our patient had been previously diagnosed in treated in another hospital. The diagnostics and proper management were performed in our department. Total BC excision with cholecystectomy and Roux-Y hepaticojejunostomy was performed. According to literature, this is the treatment method of choice in most patients.^[1]^ Liver transplantation is an important therapeutic option in patients with diffuse forms of BC (type V such as Caroli disease and Caroli syndrome). A novel surgical treatment with the use of internal biliary and pancreatic catheters was also presented. The implantation of the internal catheters into bile duct and pancreatic duct has not been described in the literature. Surgical treatment of BC is associated with low morbidity and mortality. The woman is still under close follow-up, because patients after surgery for BC require permanent and careful postoperative observation because of possibility of biliary anastomosis stricture and biliary cancer in tissue remnant.^[1]^

## 4 Conclusion

Our paper indicates that diagnosis of the abdominal cyst should be very precise in order to avoid misdiagnosis and inadequate management. The early diagnosis and proper treatment are needed in order to avoid complications associated with the cyst. The cholangiocarcinoma is the most dangerous potential complication due to dysplasia within the cyst wall as in our young female patient.

## Author contributions

**Conceptualization:** Sławomir Mrowiec, Beata Jabłońska, Magdalena Gajda, Anna Stelmach, Patryk Zemła.

**Resources:** Jan Baron, Łukasz Liszka.

**Supervision:** Sławomir Mrowiec.

**Writing – original draft:** Beata Jabłońska

**Writing – review & editing:** Sławomir Mrowiec, Beata Jabłońska, Jan Baron, Magdalena Gajda, Anna Stelmach, Patryk Zemła, Łukasz Liszka

## Abbreviations

Abbreviations: BC = biliary cyst, BT = biliary tract, CBD = common bile duct, ERCP = endoscopic retrograde cholangiopancreatography.
